# The Role of *N^6^*-Methyladenosine Modified Circular RNA in Pathophysiological Processes

**DOI:** 10.7150/ijbs.60131

**Published:** 2021-06-01

**Authors:** Mei Tang, Yonggang Lv

**Affiliations:** Mechanobiology and Regenerative Medicine Laboratory, Bioengineering College, Chongqing University, Chongqing, 400044, China.

**Keywords:** Circular RNA, *N^6^*-methyladenosine (m^6^A), Biogenesis, M^6^A-modified circRNA, Pathophysiological processes

## Abstract

Circular RNA (circRNA) is a type of covalently closed and endogenous non-coding RNA (ncRNA) with tissue- and cell-specific expression patterns generated by a non-canonical splicing event. Previous reports have indicated that circRNAs exert their functions in different ways, thereby participating in various pathophysiological processes. *N^6^*-methyladenosine (m^6^A) methylation occurs in the *N*^6^-position, which is the most abundant and conserved internal transcriptional modification in eukaryotes, including mRNA and ncRNAs. Accumulating evidences confirm that m^6^A modification also exists in the circRNA and greatly affects the biological functions of circRNA. Their dysregulated expression can be a cause of various pathophysiological processes, such as spermatogenesis, myoblast differentiation, cancer, cardiovascular disease, mental illness and so on. Understanding the role of m^6^A-modified circRNAs in pathophysiological processes may contribute to better understanding the physiological mechanisms and develop new biomarkers. This review summarizes the regulatory mechanism of m^6^A modification on circRNA metabolism and the role of m^6^A-modified circRNAs in pathophysiological processes. This article may pave the way for a better understanding of the role of epigenetically modified circRNAs in pathophysiological process.

## Introduction

Two percent of human genome transcripts are mRNAs, the rest are noncoding RNAs (ncRNAs), which can regulate genes expression in growth and development of organisms 1,2. Circular RNAs (circRNAs) are single-stranded and covalently closed endogenous ncRNAs that mainly exist in cytoplasm and have evolutionary conservation and specific expression pattern of tissue and developmental stage 3,4. Furthermore, the circular structure makes circRNAs more stable and not easily degraded by ribonucleases 3.

Although circRNAs were emerged in 1976 5,6, they were all considered as the “junk” of abnormal splicing events. Up to 2018, 30,000 circRNAs have been identified 7. Increasing studies have shown that aberrant circRNAs are related to many human diseases, such as cardiovascular disease (CVD) 8, neurological disorders 9, diabetes mellitus 10, autoimmune disease 11 and cancers 12. CircRNAs are promising in serving the biological targets for diagnosis and treatment due to their peculiarity. CircRNAs are involved in the regulation of biological processes by sponging disease-related microRNA (miRNA) 13 or proteins 14, or translating into proteins 15. Currently, it has been discovered that the post-transcriptional modification *N^6^*-methyladenosine (m^6^A), occurring in *N^6^*-position of adenosine, also exists in circRNAs and participates in the metabolism and function of circRNAs 16.

Post-transcriptional modification has become a critical regulatory factor in many physiological processes and disease progression 17. The deposition of chemically modified RNA has emerged as a basic mechanism that regulates the fate of lineages in cell transcriptome and proteome during development 18. Around 10 million peaks collected from 672 samples have been recorded in the RNA EPItranscriptome Collection database. Even though the high-resolution mapping is available, only a few amounts of modifications are mapped 20. Among those modifications, the m^6^A modification of mRNA is the most prevalent regulator in the eukaryotes 21. In addition, the dynamic and reversible m^6^A modification makes it possible to regulate the complex and delicate expression of genes. Numerous studies have indicated that m^6^A modification can respond to different physiological or pathological changes 18, 22.

Similar to mRNA, m^6^A-modified circRNAs are also installed by a multicomponent methyltransferase complex and removed by demethylases enzymes 23. Although relevant studies are still in infancy, growing evidences have shown that the biogenesis, decay, export and translation of circRNAs are affected by m^6^A modification 24. Although similar researches are still in a few, people have discovered the role of m^6^A-modified circRNA in pathophysiological processes. For instance, a groundbreaking research has summarized circRNAs with m^6^A modification are widely linked with several human diseases 23,24. Furthermore, researches demonstrated that m^6^A modification devotes to myogenesis and spermatogenesis 25,26. This review briefly addresses current studies about circRNAs and summarizes the regulatory effect of m^6^A modification on circRNA metabolism. Particularly, the role of m^6^A-modified circRNAs in the formation of some pathophysiological processes is emphasized.

## CircRNA

CircRNAs, a class of endogenous ncRNAs, are generated by variable splicing events during transcription and then form a covalently closed loop structure. Specifically, circRNAs are expressed in the different developmental stages and tissues, and play a role in plenty of important biological functions, such as affecting the development process 27, modulating immune response 28, promoting cardiac muscle repair 29, tumorigenesis or anti-tumorigenesis 30,31 and nerve injury 32. This section will introduce the biogenesis models, types and functions of circRNAs.

### Biogenesis of circRNAs

**Figure 1:**
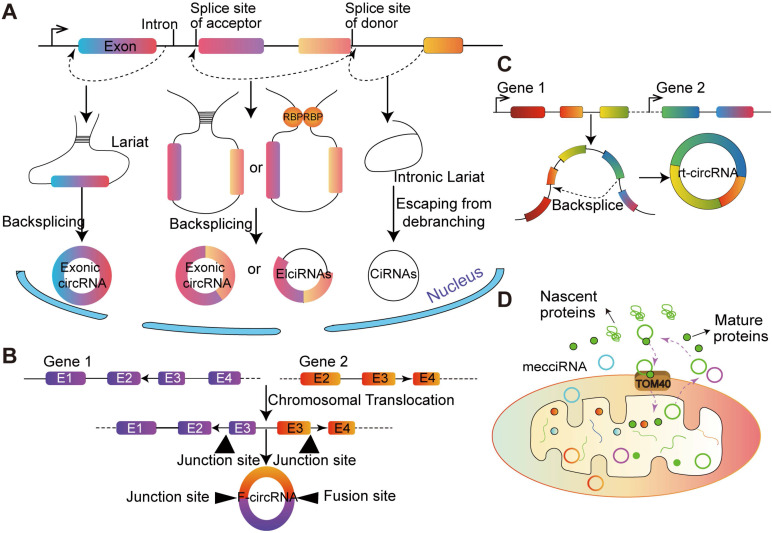
** The biogenesis of circRNAs and special types of circRNA.** (A) Lariat-driven circularization. Exon skipping events result in lariat structure, then the lariat is removed, and finally circRNA is formed. Intron-pairing-driven circularization. There are complementary sequence motifs between the introns sequences flanking the exon the base complementary forms a circular structure and the introns are removed or retained to form ecircRNAs or EIciRNAs. RBPs-pairing-driven circularization. The RBP interaction on the exon flanking sequence promotes the formation of circRNAs. CiRNAs. The ciRNAs were obtained from intronic lariat escape from debranching. (B) F-circRNA. CircRNA derived from the back-splicing of fusion-gene. (C) Rt-circRNA. Stop-codon read-through from read-through circRNA rt-circRNA. (D) MecciRNAs. CircRNAs encoded by the mitochondrial genome.

According to gene annotation, circRNA can be divided into three types according to gene annotation information: exonic circRNAs (ecircRNAs) 33, intronic circRNAs (ciRNAs) 34 and exon-intron circRNAs (EIciRNA) 1 (**Figure 1**). Although the sources of circRNAs are different, back-splicing circularization is a dominant way to produce circRNAs 35. Generally, *cis*-regulatory elements and *trans*-acting factors regulate the biogenesis of cirRNAs by controlling the splices 36.

The majorities of circRNAs are generated by protein-coding genes and contain complete exons, which implies that RNA polymerase (Pol II) participates in their transcription and spliceosome maybe involved in their biogenesis 14. Typically, circRNAs are generated from non-canonical splicing sites, and dependent on the non-canonical splicing machinery 37. A study in* Drosophila melanogaster* demonstrated that depleting one component of the spliceosome dramatically decreased the ratio of linear RNA to circRNA. The above results indicated that the processing of pre-mRNA has slowed down and nascent RNA can be used for back-splicing 14. Additionally, Kramer et al. 38 showed that depletion of splicing factors increased the level of circRNA, and multiple factors are taken additive effects, each splicing factor is not redundant in the formation of circRNA. The back-splicing hypothesis is catalyzed by canonical spliceosome machinery 39, which means the 5' and 3' ends of transcribed exons and/or introns are covalently linked. During this process, the down-stream splice site of donor exon/intron is joined to the upstream splice site of acceptor exon/intron on a pre-mRNA molecule 40 (**Figure 1**).

Multiple splice variants of circRNA transcripts can be generated from a single gene, depending on the exons selected during the back-splicing 41. In the process of biogenesis, part of the RNA is transcribed from pre-mRNA, and exons are skipped as RNA folds. These structural changes generate some areas called lariat structures, in which discontinuous extrapolation zones initially become very close as the interior changes. CircRNA is formed after removing the subsequence from the lariat structure. This splicing process is called as 'lariat-driven circularization'. Due to the internalized feedback sequences of the former mRNA, the complementary pairing of introns on both sides forms circRNA. Another lariat-driven circRNA is ciRNA, whose formation processes depend on consensus RNA motifs near 5' splice site and branchpoint 42. Studies in *Homo sapiens* have identified hundreds of ciRNAs 37. CiRNAs are largely restricted to the nucleus and regulate gene expression in a *cis* manner.

General speaking, endogenous human circRNAs comprise two or three exons, or even more 42, and back-splicing requires *cis*-elements in intron flanking circularized exons 34. Mutagenesis results indicate that the circularization of exons is dependent on the presence of short (~30- to 40- nucleotide) inverted repeats, such as the Alu element 43. Besides, hundreds of *trans*-regulators, such as RNA-binding proteins (RBPs) and miRNA-containing ribonucleoprotein complexes, control the gene expression at the posttranscriptional level in eukaryotes 44. For example, circRNAs can be produced when the synthetic Quaking (QKI)-binding sites insert into introns 45. RBPs in different systems and organisms can also regulate exon circularization, such as adenosine deaminases acting on RNA, nuclear factors NF90/NF110, fused in sarcoma, DExH-Box helicase 9, serine/arginine-rich proteins and epithelial splicing regulatory protein 1 46.

### Types and characteristics of circRNAs

Generally, circRNA can be divided into three types, and some specific sources of circRNA have recently been discovered (**Figure 1**). It has been demonstrated that fusion-gene is the driver in many tumors, including leukemia 47 and lung cancer 48,49. CircRNAs derived from fusion-gene (F-circRNA) (**Figure 1B**), such as *PML/RARα* and *MLL/AF9* fusion genes, have a carcinogenic effect *in vivo* model 45. F- circEA-2a, derived from *EML4-ALK-v3b* with 'AA' motif at the junction site, promotes the migration and invasion of non-small cell lung cancer cell and acts as a diagnostic marker for lung cancer 48,49. Additionally, Wu et al. 50 demonstrated that F-circrSR1 and F-circrSR2 originated from the same fusion gene solute carrier family 34 member 2 and reactive oxygen species proto-oncogene 1 (*SLC34A2-ROS1*), both of which promoted the migration of lung cancer cells, but did not affect cell proliferation.

Commonly, mRNA translation will not terminate until meet the stop codon. However, in some unconventional conditions, the ribosome goes past the stop codon and continues translating into an otherwise untranslated region (UTR) of a transcript, which is known as 'stop-codon read-through' 51. It is reported that the transcriptional read-through process is related to the formation of circRNA. Genes that interfere with the regulation of transcriptional termination may contribute to the production of transcriptional read-through products and promote the generation of circRNA from downstream gene sources 52. Moreover, based on more than 2,000 human tumor specimens from different tissue sources, and a novel type of circular RNA transcript was uncovered, termed as read-through circRNA (rt-circRNA) (**Figure 1C**) 12. Lately, Liu et al. 53 reported that the mitochondrial genome of humans and mice encode hundreds of circRNAs. These mecciRNAs (**Figure 1D**) act as a molecular chaperone to facilitate nuclear-encoded proteins entry into mitochondria. This is the first report of circRNA encoded by mammalian mitochondrial genome.

### Functions of circRNAs

#### Modulate miRNA functions

Only small parts of the biological function of circRNAs have been investigated, one of the most typical mechanisms is to act as a miRNA sponge 13. Many circRNAs have specific binding sites with miRNAs, which decreased miRNA activity and increase miRNA-target gene activity. Piwecka et al. 54 found that miR-7 expression was significantly decreased in CDR1as knockout mice, while some targets of miR-7 increased in CDR1as-knockout mice brain. Recently, it was demonstrated that Cyrano binds and targets miR-7 for degradation in CRISPR-Cas9 engineered mice 55. This effect of Cyrano on miR-7 indirectly modulated the degradation of CDR1as by miR-671, which reveals a molecular regulatory network composed of non-coding RNAs.

Many studies illustrated that circRNAs participate in pathological conditions by sponging miRNAs. For example, high expression of circRNA-000284 in cervical cancer tissues promotes the migration, proliferation and invasion of cancer cells through the sponge of miR-506 56. CircRNAs, hsa_circ_0001564 and hsa_circ_0009910 can facilitate osteosarcoma cell proliferation, migration and invasion by targeting miR-29c-3p and miR-449a, respectively 57,58. CircSEPT9 activates the LIF/STAT3 pathway by competitively binding to miR-637 in triple-negative breast cancer 59. Jost et al. 60 successfully designed an artificial circRNA that can sponge miR-122 in hepatitis C virus (HCV) cells, thereby preventing the formation of HCV viral proteins and alleviating hepatitis C. In neonatal mouse ventricular cardiomyocytes, circRNA_000203 aggravated cardiac hypertrophy *via* specific adsorption miR-26b-5p and miR-140-3p 61. Notably, most previously identified mammalian circRNAs are expressed at low level and do not have miRNA binding sites. Thus, circRNAs may not only act as miRNA sponges 35. The latest findings showed that hsa_circ_0008558 (circLONP2) can regulate the intercellular miRNAs maturation and metastasis, accelerating the metastasis of colorectal cancer cell to other organs 62.

#### Regulate parental gene transcription

Some scholars have pointed out that circRNAs also can participate in gene transcription regulation. *ANRIL* is an antisense ncRNA in cyclin-dependent kinase 4 inhibitor (INK4) locus and is found in melanoma 63. Increased expression or mutation of *ANRIL* is associated with coronary atherosclerotic heart disease and atherosclerosis 64. *ANRIL* also inhibits the transcription of the INK4 and its alternative reading frame (ARF) by interacting with the polycomb group complex. Burd et al. 65 speculated that the circular *ANRIL* (*cANRIL*, product of *ANRIL* back-splicing) may regulate the expression of INK4/ARF. Most ciRNAs are abundant in the nucleus and have few miRNA target sites. Importantly, knockdown of ciRNAs hinders the corresponding parent gene transcription. Ci-ankrd52 (ciRNA from the gene *ANKRD52*) deposits in the transcription initiation region of the gene, associates with the Pol II extension mechanism, and promotes the function of RNA Pol II 34.

In addition, EIciRNAs, such as EIciPAIP2 and EIciEIF3J, promote host gene transcription by interacting with the U1 small nuclear ribonucleoproteins (snRNP) in RNA-RNA conjunction to form the EIciRNAs-U1 snRNP complex 66. In *Arabidopsis*, exon 6 of the *SEPALLATA3* gene cyclizes and forms an R-loop structure of RNA-DNA hybrid complex by circRNA strongly binding the cognate DNA locus of host gene. R-loop structure inhibits the transcription of this region and allows the exon skipping 67. Subsequently, this circRNA functions by modulating the alternative back-splicing of parental transcript 68. All these functions suggest that circRNA can bind to genomic DNA to regulate alternative splicing and *cis*-modulate diseases genes.

#### Interact with proteins

Some circRNAs have one or more RBPs sites, which can be used as proteins sponges to isolate and inhibit the biological function of proteins. For example, cADR1as and sex-determining region Y circRNAs can bind to miRNA response factor Argonaute to be degraded 68,69. MBL circRNA (circMbl) flanking intron has many muscleblind (MBL) binding sites. Studies have shown that MBL is related to the biological synthesis of circMbl. When MBL protein is redundant, it will reduce the level of its own mRNA by promoting the generation of circMbl 14. CircPABPN1 is derived from the poly (A) binding protein 1 (*PABPN1*) gene, and the Hu-antigen R (HuR) regulates the abundance of PABPN1 instead of circPABPN1. Interestingly, circPABPN1 and *PABPN1* competes for the HuR protein binding sites 70.

Furthermore, circRNAs participate in a variety of physiological processes by interacting with proteins. Du et al 71 found circ-Foxo3 could be retained in the cytoplasm through interactions with anti-aging and anti-stress protein-related factors, such as inhibitors of DNA-binding 1 (ID-1) protein, focal adhesion kinase and hypoxia inducible factor 1α, thereby hindering the progress of the corresponding resistance. By binding to mouse double-minute 2 (MDM2) and p53, circFoxo3 can promote the ubiquitination of p53 induced by MDM2, leading to the overall degradation of p53 72. Recently, some studies have found that two or more proteins are assemble into greater protein complexes, such as enzymes and their substrates, in which circRNA can serve as protein scaffolds 73. For example, circ-Foxo3 forms a ternary complex with p21 and CDK2, which can inhibit the function of CDK2 and block cell-cycle process 74. Another example is the acute myeloid leukemia (AML)-associated circMYBL2. This circRNA regulates the translation efficiency of oncogene FMS-like tyrosine kinase-3 (FLT3) by recruiting PTBP1, resulting in progression of FLT3-internal tandem duplication AML 75. Designing an exogenous circRNA to target cancer, diabetes, or other disease-related protein may be useful for medical treatments.

#### Translate into proteins

Although circRNAs have been well-described as miRNA sponges, many of them do not contain miRNA capture sites. Researches indicated that many circRNAs are generated from exons localized in the cytoplasm, suggesting that they can be translated into peptides by loading into ribosomes 33. The genome of hepatitis delta (D) virus (HDV) has been identified with a natural translatable circRNA. The core circRNA of HDV encoded the HDV antigen, which plays critical role in the hepatitis disease development 76. Guo et al. 77 found that circRNAs could be translated in human osteosarcoma U2OS cells, while most circular isoforms are far less efficiently compared to their linear isoforms. Because circRNAs do not have a 5'cap structure and cannot be translated into protein through cap-dependent mechanisms. Another way of RNA translation is to use the internal ribosome entry site (IRES) sequence as an internal ribosome entry sites for translatable circRNAs 78. Artificial circRNAs with a RES can be translated *in vitro* or* in vivo* 79-81. The covalently closed circular RNA is found in rice yellow mottle virus, which possesses an internal ribosome entry site and utilizes two (or three) open reading frames (ORFs) that directly translate into a 16-kDa highly basic protein 82.

Although many circRNAs have ORFs and with an upstream IRES elements, 46 of these are translated into corresponding proteins according to mass spectrometry 83. So far, only circular RNA zinc finger protein 609 (circ-ZNF609), circMb1, circ-FBXW7, circPINTTaxon2, circ-SHPRH and circβ-catenin have the coding ability in related studies. Circ-ANF609 is related with heavy polysomes and can be translated in an IRES-dependent mode, even though the translation activity of the circular template is much lower than the linear counterpart template 84. Furthermore, decreases of circ-ZNF609 expression cause damage to the proliferation of human and mouse myoblasts, and the translation of circ-ZNF609 can be significantly induced by heat shock. Circ-Mb1 shares the same start codon with the host RNA, encoding a peptide in a cap-independent manner in *Drosophila*, starvation can regulate the circ-Mb1 production and/or stability depending on 4E-BP and FOXO proteins 15.

Additionally, circ-FBXW7 85, circ-SHPRH 86, 87 and circPINTTaxon2 88 can produce FBXW7-185aa, PINT87aa and SHPRH-146aa, respectively. All of them can inhibit human glioblastomas. Circ-FBXW7 is extremely abundant in the brain, and IRES drives a 21-kDa protein named FBXW7-185aa. FBXW7-185aa directly interacts with de-ubiquitinating enzyme USP28, which can stabilize the oncoprotein c-Myc by inhibiting FBXW7α (the most abundant isoform of the *FBXW7* gene) activity. FBXW7-185aa can inhibit proliferation and cell cycle of U251 and U373 cell lines by this way 85. Two reports have successively reported that circ-SHPRH (originate from the sucrose non fermenting 2 histone linker PHD RING helicase (*SHPRH*) gene) employs overlapping genetic codes to encode a 17-kDa peptide named SHPRH-146aa 85,86. Theoretically, SHPRH-146aa protects full-length SHPRH from degradation by ubiquitin proteasomes. E3 ubiquitin protein ligase SHPRH increases its anti-tumor function by targeting proliferating cell nuclear antigen. Finally, circPINTTaxon2 is generated from long intergenic non-protein-coding RNA p53-induced transcript gene and encodes a peptide named PINT87aa, which can inhibit multiple oncogenes though interacting with polymerase associated factor complexes and act as a tumor suppressor 88. Polymerase associated factor complex is involved in the recruitment of RNA Pol II and the transcriptional elongation of downstream genes 89. Circβ-catenin encodes a 370-amino acid β-catenin isoform by IERS, which prevents β-catenin from phosphorylation and degradation by antagonizing glycogen synthase kinase 3β and subsequently promotes tumor growth through activating the Wnt pathway 90. The information about circRNA-encoded proteins is scattered in many published papers, which is not conducive to further exploration on circRNA translation. Liu et al. 91 presented an ncEP database, which recorded all published articles containing proteins or peptides encoded by ncRNAs. These discoveries imply that the coding potential of circRNAs has been largely disregarded, which may help to deepen our understanding of circRNAs.

#### Pseudogenes derived from circRNAs

Around 19,000 conserved pseudogenes have been revealed in human by sequencing efforts and their transcription patterns display tissue and pathological condition specificity 92. Researchers developed computational pipeline to identify circRNAs-originated pseudogene, called CIRCpseudo, to determine whether stable circRNAs can be retrotranscribed and integrated into the host genome. Dozens of circRNAs-derived pseudogenes have been identified 93. Among them, at least 33 circRNAs at the ring finger and WD repeat domain 2 (RFWD2) locus-derived pseudogenes (circRFWD2) were found in different mouse strains, characterized by the exon 6-exon 2 anchor in a reversed order. The pseudogene derived from circSATB1 can specifically bind to CCCTC-binding factor and/or Rad21-binding sites in several mouse cell lines to regulate gene expression. In addition, the insertion of retrotransposed circRNAs into the genome changes the composition of genomic DNA and regulates the potential for gene expression 93.

#### CircRNA acts as a ribozyme

Satellite virus, viroid and HDV RNA exist in a circular form, and RNA is part of a replication ring that contains self-dividing sequences (ribozymes). Through the research of the classical type I hammerhead ribozyme (HHR) motif in diverse metazoans genomes, a novel and conserved small ribozyme has been found to efficiently synthesize circular RNA 94. A systematic analysis of the genomes of cnidaria (a coral), mollusca (a mussel) and chordata (a salamander) has revealed that there are abundant type I HHR motifs in the DNA tandem repeats of their genomes, with a length of about 170-400 nt, mostly in the form of linear and circRNAs. These modifications confirmed the existence of novel natural pathways for circRNA biosynthesis through a conserved autocatalytic RNA in metazoan.

## M^6^A modification

Epigenetic modifications are involved in cell fate and respond to environmental stimuli by regulating gene expression 95. These modifications are implicated in the development of organism 18,96,97. While the role of epitranscriptomic modifications in gene regulation is scarcely acquainted. Recently, considerable studies have revealed the role of post-transcriptional RNA modifications in modeling an epitranscriptomic landscape of gene expression 23,98. RNA modifications tend to deposit in highly abundant RNA species to regulate RNA splicing, transport, translation and turnover 99.

There are more than 170 known RNA modifications, such as m^6^A, *N^1^*-methyladenosine, inosine, 5-methylcytidine, 5-hydroxymethylcytidine, pseudouridine, *N^6^*,2′-O-dimethyladenosine, *N^4^*-acetylcytidine, *N^7^*-methylguanosine, 8-oxoguanosine and 2′-O-methyl 100. Among them, m^6^A is the most abundant modifications of mRNAs found in all eukaryotes 22. Since around 20-40% of mammalian transcripts are m^6^A methylated, and the methylated mRNAs tend to have multiple m^6^A modifications 21. Furthermore, recovering the mechanisms of deposition, removing and recognition RNA modifications (more preferably known as writers, erasers and readers, respectively) have helped to understand the fates of those post-transcriptional modifications in cellular processes, such as cell growth, body development and diseases.

### Regulators of m^6^A

**Figure 2:**
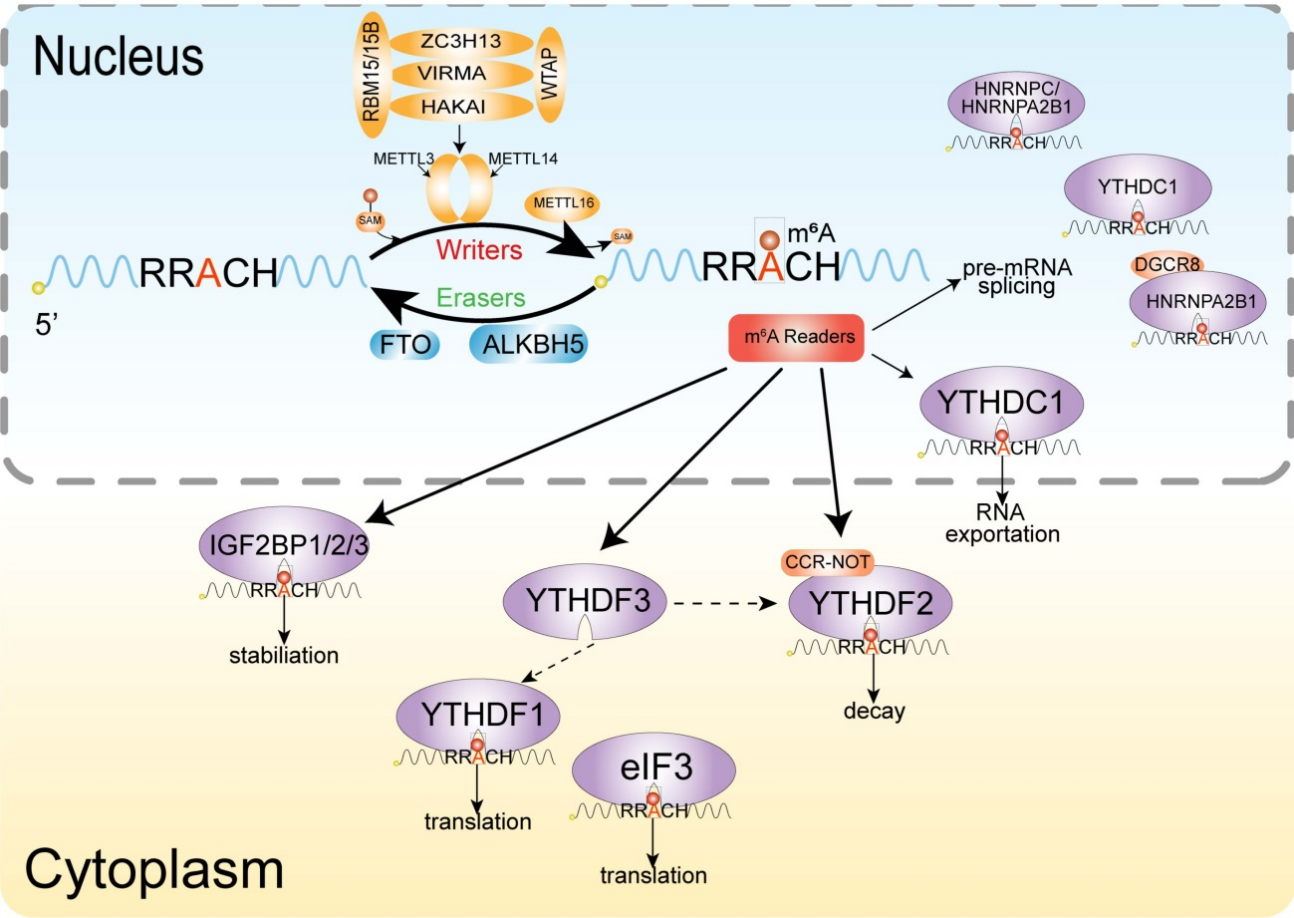
** Mechanism of m^6^A.** The dynamic modification of m^6^A modification is installed by the methylransferase complex (writers) composed of METTL3, METTL14, WTAP, KIA1429, ZC3H13 and RBM15/RBM15b, and removed by RNA demethylases (erasers) FTO and ALKBH5. Methyl-specific binding proteins (readers) in are the YTH family, which can achieve the biological functions of m^6^A modification.

M^6^A is similar to DNA and histone methylation, a dynamic and reversible event 101, and the methyltransferases and demethylases coordinately regulate the deposition and decay of m^6^A, also named with m^6^A writer and m^6^A eraser respectively (**Figure 2**). M^6^A is installed by a multiprotein methyltransferase complex (MTC, also termed m^6^A 'writer'). The core of MTC is a heterodimer core catalytic subunit composed of methyltransferase-like 3 (METTL3) and methyltransferase-like 14 (METTL14) 102-104. There are many other accessory subunits, including wilms tumor 1 associated protein (WTAP) 105, vir-like m^6^A methyltransferase-associated (also known as Virilizer or KIAA1429) 106, RNA binding motif protein 15 107, cbl proto oncogene-like protein 1 (also known as Hakai) 108 and zinc finger CCCH domain-containing protein 13 (ZC3H13) 107. METTL3 is the catalytic subunit and METTL14 acts as the RNA-binding platform. Moreover, the METTL3 homolog methyltransferase-like 16 (METTL16) can catalyze the m^6^A onto the U6 small nuclear RNA (snRNA) and some structured RNAs 110. In addition to the above cofactors, other catalytic subunits join the methyltransferase complex, which is designed for precise post-transcriptional regulation 85. Two demethylases, fat mass and obesity-associated protein (FTO) and α-ketoglutarate-dependent dioxygenase alkB homolog 5 (ALKBH5), can remove the m^6^A modification, acting as erasers 101,111.

The m^6^A modification realizes its biological function specific recognition and binding of RNA binding proteins (readers), thereby affecting RNA fate by regulating RNA metabolism. Several proteins or complexes that specifically recognize m^6^A sites have been identified, including YT521-B homology (YTH) domain-containing protein 112, eukaryotic initiation factor 3 96, IGF2 mRNA binding proteins (IGF2BP) families 113 and heterogeneous nuclear ribonucleoprotein protein family. The YTH domain can recognize m^6^A through a conserved aromatic cage and another two proteins FMRA, LRPPRC 'read' this modification 114.

### Characteristics and functions of m^6^A

M^6^A modification exists in different types of mammalian cell, including blood, liver, neuronal and muscle cells and so on. The deposition of m^6^A modification affects the process of cell metabolism, transcription, splicing, translation, degradation and localization.

The distribution of m^6^A modification on mRNA is sequence-specific and often appears on a consensus RNA motif of RRACH (R = G or A; H = A, C or U), coding sequence, 3'UTRs, especially near stop codons 115. M^6^A modification can cause changes in RNA secondary structure by affecting RNA pairing, regulate RNA stability and its interaction with proteins 116,117. M^6^A is also highly enriched in the exon region near the splicing site, and has significant spatial overlap with the splicing factor SRSF1 and SRSF2 RNA binding sites. Experimental studies have revealed that m^6^A-modified exons tended to be retained during splicing 118. Ke et al. 119 illustrated the molecular mechanism of m^6^A-modified exons being retained during the splicing process: m^6^A binding protein YTHDC1 promotes its retention by promoting SRSF3 and suppressing SRSF10 in combination with m^6^A-modified exons. M^6^A also shows a tendency of enrichment in the last exon of a transcript, which may be related to the regulation of the alternative polyadenylation of mRNA. The unstable pairing of m^6^A-U causes the melting of double-stranded RNAs and transformation of secondary structure.

### M^6^A methylation on circRNAs metabolism

**Figure 3:**
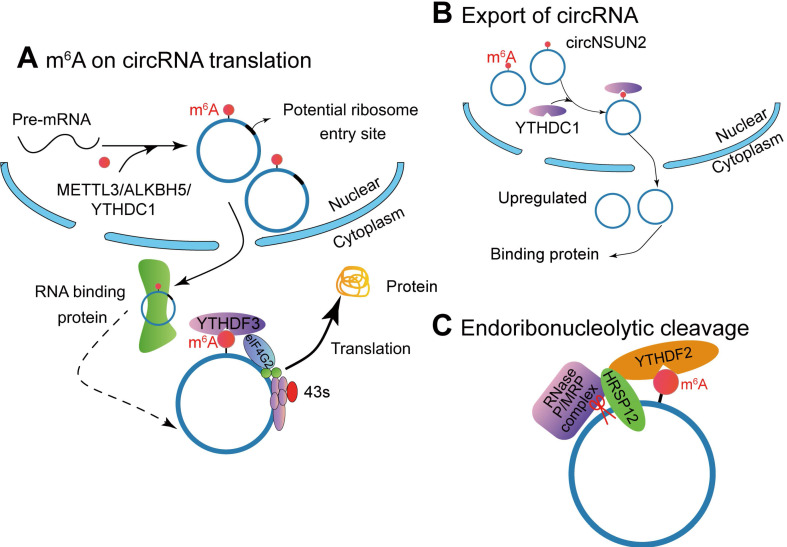
** M^6^A modification affects the function of circRNAs.** (A) METTL3, ALKBH5 and YTHDC1 involve in the biogenesis of circRNA, and m^6^A-modified circRNA can be translated into proteins in a cap-independent manner with YTHDF3 and translation initiation factors eIF4G2 and eIF3A. (B) The m^6^A reader YTHDC1 promotes circRNA cytoplasmic export. (C) The YTHDF2-HRSP12 mediated RNase P/MRP complex binds and degrades circRNAs.

Chemical covalent modification of nucleotides molecules is a ubiquitous life process and many studies have illustrated chemical modifications in DNA and RNA. There have been identified more than 100 types of chemical modifications of mRNAs, rRNAs, snRNAs and snoRNAs have been identified in organisms. It has been reported that linear mRNA and lncRNA have m^6^A modification. Currently, m^6^A modification has been identified and characterized in circRNA, but the potential regulatory mechanism has not been fully elucidated 120. The researches on circRNAs with m^6^A-modification, and the function of these m^6^A-modifications are described in **Figure 3**.

#### M^6^A on circRNA translation

Studies have shown that circrRNAs have coding potential. Yang et al. 16 described a new mechanism that circRNA with m^6^A residues can be translated in a cap-independent manner (**Figure 3A**). These translatable circRNAs contain a large number of m^6^A consensus motifs (13% of total circRNAs), and a single m^6^A site is enough to initiate circRNA translation. The translation of m^6^A-modified circRNA depends on IF eIF4G2 (non-canonical eIF4G) protein and m^6^A reader YTHDF3. The m^6^A-driven translation is reversible, because methyltransferase METTL3/14 can increase the translation efficiency, while inhibited by the demethyla FTO. Moreover, mRNA with m^6^A modification in its 5'UTR can be translated under specific cellular stress (such as amino acid starvation) and through 5'cap-independent way to regulate the translation 121. Here, they proved that m^6^A modification continuously activates the translation of circRNA with continuation of heat shock stimulation or overexpression of METTL13/14. They also authenticated 250 circRNAs associated with polysome. Puromycin can significantly reduce the amount of circRNAs, which indicates that circRNAs with polysomes may be actively translated 16. However, how m^6^A modified circRNA is generated has not been clarified yet.

Tang et al. 26 found that m^6^A modification could promote the formation of ORF-carrying circRNA by studying the development process of male germ cells in mice. Sequencing analysis during spermatogenesis has found that a large number of circRNAs are generated while the corresponding linear mRNA decreased. Some of the circRNAs often carry high levels of m^6^A modification on both sides of the reverse junction point, and m^6^A is often enriched around the ORF start and stop codons of the mRNA (**Figure 3A**). Interestingly, Tang et al. 26 found that nearly half of these circRNAs carry longer ORFs whose start codons are modified by m^6^A to bind ribosomes. Hundreds of peptides encoded by these circRNAs were detected by liquid chromatography and mass spectrometry. This discovery indicates that m^6^A can mediate the generation of circRNA and reports a new mechanism that relies on circRNA to achieve the stable expression of protein products after linear RNA deletion. Di Timoteod et al. 121 further demonstrated this point by taking circ-ZNF609 as a study case. The generation of circ-ZNF609 modified by m^6^A is regulated by METTL3/YTHDC1, and the recognition of m^6^A site by YTHDF3 and eIF4G2 promotes circ-ZNF609 translation.

#### Endoribonucleolytic cleavage of circRNA

M^6^A is involved in regulating multiple steps of mRNA modification, including stability, and the degradation of mRNA containing m^6^A modification is mediated by YTHDF2 (**Figure 3B**). In detail, it depends on the presence of the adaptor protein heat-responsive protein 12 (HRSP12)-binding site in the messenger ribonucleoprotein. If it exists, deadenylation pathway mediated by CCR4/NOT complex is activated, leading to deadenylation and decay of m^6^A-containing mRNAs 122 or endoribonucleolytic cleavage pathway by the YTHDF2-HRSP12-RNase P/mitochondrial RNA-processing (MRP) (endoribonuclease) complex causing degradation of YTHDF2-bound RNAs 123. Comparing to mRNA, circRNAs have a covalently closed loop and do not have a 3' polyadenylated tail, they are naturally more stable than their homologous linear RNAs in both intracellular and extracellular environments and their degradation can be avoided 124. Therefore, circRNAs can only be degraded by endoribonucleolytic cleavage. Park et al. 123 demonstrated that circRNAs containing m^6^A can be decayed through YTHDF2-HRSP12-RNase P/MRP-mediated endoribonucleolytic cleavage. The abundance of circRNAs containing m^6^A increased after a component of RNase P/MRP was down-regulated 123.

#### Promote the nuclear export of circRNA

Following biogenesis, most circRNAs are efficiently exported to cytoplasm. However, the intron-containing circRNAs accumulate in the nucleus 68,69. Some nondividing cells (e.g. neurons) highly express circRNAs. Therefore, the transport process regulates the localization of circRNAs, depending on nuclear envelope breakdown during mitosis. Based on RNA interference screening in *Drosophila*, the interference of Hel25E significantly led to the enrichment of circRNA in the nucleus. Further identification showed that Hel25E was an important regulator of post-transcriptional nuclear export of circRNAs. In human cells, circRNAs are transported from the nucleus in a transcript-length-dependent manner to the cytoplasm via Hel25E homogenous proteins: ATP-dependent RNA helicase DDX39A (also known as nuclear RNA helicase URH49 or URH49) and spliceosome RNA helicase DDX39B (also known as DEAD box protein UAP56 or UAP56) 125.

Knockout of m^6^A demethylase ALKBH5 accelerates the nuclear export of mRNA (**Figure 3C**) 126. Mechanism studies have proved that YTHDC1 interacts with splice factor SRSF3 to recruit NXF1, thereby promoting the nucleation of m^6^A mRNA 127. Chen et al. 128 found that m^6^A-modified circRNA NOP2/Sun RNA methyltransferase family member 2 (NSUN2) locus (circNSUN2) exports from the nucleus to cytoplasm by binding to YTHDC1. Then cytoplasmic circNSUN2 interacts with RBP and insulin-like growth factor 2 mRNA-binding protein 2 (IGF2BP2) to promote the stability of high mobility group AT-hook 2 (HMGA2) transcript 128. Compared with paracancerous tissues, circNSUN2 is highly expressed in colorectal cancer tissues during the progression of lymph node metastasis and liver metastasis in colorectal cancer, and the expression level of circNUSN2 gradually increases. Further, *in vivo* nude mouse metastasis and *in vitro* cell function experiments show that circNSUN2 promotes the metastasis of colorectal cancer tumours. This circRNA binds to the m^6^A binding protein YTHDC1 in the nucleus, and YTHDF1 regulates the nuclear localization of circNSUN2 in an m^6^A-dependent manner. The cytoplasm of circNSUN2 can bind to the RNA-binding protein IGF2BP2 and the downstream *HMGA2* (a member of the high motility group, HMG) mRNA to form a circNSUN2/IGF2BP2/*HMGA2* RNA-protein ternary complex, which promotes the stability of *HMGA2* mRNA. Ultimately, m^6^A modified circNSUN2 promotes liver metastasis of colorectal cancer tumors by accommodating the stability of HMGA2 mRNA 128. It is suggested that the interacting proteins can help to discover inner mechanism of circRNAs.

## m^6^A-modified circRNA associated with pathophysiological processes

With the gradual understanding of the structure and function of m^6^A modification and circRNA, researchers have investigated the role of m^6^A-modified circRNAs in physiological and pathological processes. Additionally, many evidences were showed that m^6^A-modified circRNA are related with the progress of various cancer 24, 128-132, immune response 133, CVD 134, mental illness 135, myoblast differentiation 25, spermatogenesis 26 and so on. The pathophysiological processes regulated by m6A-modified circRNAs are summarized in here.

### m^6^A-modified circRNA in pathological processes

Although most m^6^A-modified circRNAs are thought play a role in pathological processes, the function of m^6^A modification on circRNA is unknown. **Table 1** lists the proposed mechanism and functions of m^6^A-modified circRNAs, and is discussed below.

#### Cancers

Wealth of recent studies have revealed that circRNA and m^6^A modification exert a regulatory function in different diseases, and many of them highlighted a role in various cancer 17,136. Cancer is a leading cause of mortality worldwide 137. Based on the structure and function of m^6^A-modified circRNA, researchers have found that they play an important role in the progression, proliferation and metastasis of cancers, such as colorectal cancer 128, gastric cancer 129, hepatocellular carcinoma (HCC) 130,131 and poorly differentiated gastric adenocarcinoma 132.

A study on gastric cancer (GC) found that knockdown of circRNA poliovirus receptor-related 3 (circPVRL3) significantly promoted cell proliferation 129. The internal ribosomal entry sites, open reading frame and m^6^A modification are present on circPVRL3, indicating that circPVRL3 has coding potential. Overall, this study pointed to the m^6^A-modified circPVRL) in the carcinogenesis 129. 30 upregulated circRNAs and 35 downregulated circRNAs were evaluated in poorly differentiated gastric adenocarcinoma (PDGA) by circRNA microarray. The level of m^6^A was positive relative with circRNA in PDGA, which indicated that m^6^A-modified circRNA plays a role in GC progression 130.

Liver cancer has a high morbidity and mortality rate worldwide 137. In HCC, circ_KIAA1429 (originate from KIAA1429) can accelerate the progress of HCC through the m^6^A-YTHDF3-Zeb1 mechanism, and may be a potential target for the HCC therapy 131. In addition to modifying m^6^A modification process, circRNA can also affect the stability of circRNA via changing the methylation states. In HCC, circRNA-SORE acts as a competitive endogenous RNA, which induces sorafenib resistance by competitively activating the Wnt/β-catenin pathway by adsorbing miR-103a-2-5p and miR-660-3p. They found that the increasing m^6^A level of circRNA-SORE can maintain its stability and promotes the development of drug resistance 132. This paper expounds the new mechanism of drug resistance in targeted therapy of liver cancer and provides new clues for finding new therapeutic targets for advanced liver cancer patients.

Lung cancer is the leading cause of cancer death in men 138. Recently, a study indicated that circNDUFB2, derived from NADH: ubiquinone oxidoreductase subunit B2, is frequently downregulated in non-small cell lung cancer (NSCLC), and negatively correlated with the malignant characteristics of NSCLC. CircNDUFB2 acts as a scaffold of tripartite motif containing 25 and IGF2BPs to form a ternary complex, which promotes the degradation of IGF2BPs and inhibits the growth and metastasis of NSCLC cells. Furthermore, m^6^A-modified circNDUFB2 can enhance this effect 139. In addition, circNDUFB2 recognized by RIG-I can active RIG-I-MAVS signaling cascades, thereby recruiting immune cells into the tumor microenvironment. Apart from enhancing IGF2BPs stability, the down-regulated circNDUFB2 in NSCLC also leads to immune evasion and promotes tumor progression 139.

In addition, some m^6^A-modified circRNAs have not yet clarified their unctions. In esophageal squamous cell carcinoma (ESCC), circ-SLC7A5 was significantly up-regulated in the plasma of ESCC patients and high levels of circ-SLC7A5 are correlated with shorter survival time. Bioinformatics predicts that circ-SLC7A5 has m^6^A modification structure with translation potential 140. Zhou et al. 32 developed a computational pipeline together with experimental results, which revealed m^6^A circRNAs are widely spread in circRNAs (they accounts for 41% of the total circRNAs) and are significantly different between human embryonic stem cells (hESCs) and HeLa cells. Moreover, even if circRNAs are originated from the same mRNA, they cannot be detected in different cell types. Up to 65% of m^6^A-modified circRNA derived from HeLa cells cannot be detected in hESCs. Furthermore, about 41% of HeLa-generated circRNA do not appear in hESCs. Zhou et al. 32 also found some other features of m^6^A-modified circRNA: 1) the distribution of m^6^A modification sites on circRNAs and corresponding mRNAs are different; 2) the parental exons of m^6^A circRNAs are relatively greater than those of non-methylated circRNAs; 3) circRNAs share the same m^6^A modification enzymes with linear RNA; 4) the host mRNA is unstable. The m^6^A expression level is negatively associated with circRNA expression. This article found that circRNA has a cell-specific m^6^A modification state, which can be used to diagnose diseases.

#### Immune response

CircRNA is automatically spliced and cyclized after *in vitro* transcription to transfected cells. Unexpectedly, this type of circRNA can induce the activation of innate immune response and inhibition of the RNA virus infection process by retinoic acid-inducible gene-I (RIG-I) 134. Endogenous circRNA with m^6^A modification can suppress innate immune responses by inhibiting RIG-I activation 141. When cells are stimulated by polyinosinic acid-polycytidylic acid or infected by viruses, the endonuclease RNase L activates and degrades circRNA molecules. Protein kinase (PKR) is released and activates the downstream antiviral mechanism systematic lupus erythematosus (SLE). The expression of circRNA in the peripheral blood mononuclear cells (PBMC) of SLE patients decreased and the activity of PKR increased. However, overexpression of circRNA in PBMC derived from SLE patients can reduce the activity of PKR, which may be helpful for the treatment of autoimmune diseases such as SLE 134.

Chen et al. 134 found that the lack of sample purity might be caused by the non-specificity of the substituted linear RNA with 5' phosphate effect. Thus, Chen et al. 134 did some other experiments to show that their research system is reliable and produced a novel mechanism. The m^6^A reader YTHDF2 may be reason for the RNA degradation 19, 24. Studies have shown that m^6^A modification controls immune responses 142. CircE7 was modified by m^6^A and translated to produce E7 oncoprotein 143. CircE7 exists in TCGA RNA-Seq data of human papillomavirus (HPV)-positive cancer cells and in cell lines where only free HPV is present. These results prove that virus-derived that can encode proteins has biological functions and is related to certain HPV transformation characteristics 144.

Wesselhoeft et al. 144 found that exogenously synthesized circRNA transfected into cells did not induce toll-like receptor/RIG-I-mediated innate immune response, and circRNAs can more effectively translate into proteins in mouse tissues. Deposition of m^6^A modification in circRNA can recruit and bind YTHDF2, inhibit the activation of RIG-I/K63-Ubn/circRNA complex and do not induce the innate immune response 144. Collectively, these finding proved that m^6^A modification of circRNAs is a key regulator in circRNA related immune responses.

#### CVD

CVD causes 17.5 million annual deaths and increases the burden of public health 145. Cell death is one of the critical pathological mechanism of atherosclerosis (AS). IFN regulatory factor (IRF)-1 has been demonstrated play a critical role in regulating cell death of AS. The relative RNA expression level of hsa_circ_0029589 in macrophages of acute coronary syndrome decreased, while the m^6^A level of hsa_circ_0029589 and the m^6^A METL3 were significantly increased 146. In addition, the overexpression of IRF-1 inhibited the expression of hsa_circ_0029589 in macrophages, and simultaneously induced the expression of m^6^A and METL3. Overexpression of hsa_circ_0029589 or inhibition of METTL3 can significantly increase the expression of hsa_circ_0029589 and reduce macrophage apoptosis 146. Increased m^6^A abundance in hypoxia mediated pulmonary hypertension (HPH) reduces the total circRNAs abundance in hypoxia *in vitro* 147. M^6^A affects the co-expression network of circRNA-siRNA-mRNA during hypoxia. Specifically, the m^6^A-modified circXpo6 and circTmtc3 may be used as HPH biomarkers due to their different enrichments in specific pathological condition 146. The difference of m^6^A circRNAs in cells or tissues suggests that they may be involved in progression of CVD or act as a pathological marker.

#### Age-related cataract

Age-related cataract (ARC) is the leading cause of world blindness, which causes ~50% blindness worldwide 148. Few circRNA has been identified in cataract. For instance, circKMT2E sponges miR-204 to regulate the pathogenesis of diabetic cataract 149. Total circRNA expression level was decreased in cortical of ARC (ARCC). Compared with non-m^6^A modified circRNAs, the expression of highly abundant m^6^A circRNAs were mostly reduced. Bioinformatics analysis predicted that ALKBH5 was significantly upregulated in lens epithelium cells (LECs) of ARCC among the five major methyltransferases. Those results indicate that M6A modification of circRNAs may be associated with LEC lesions by regulating genes/pathways related to the onset of ARC 150.

#### Major depressive disorder

Major depressive disorder (MDD) is a severe mental disorder with high incidence 151. Currently, several studies have demonstrated circRNAs play a role in MDD. CircDYM is originated from *DYM* gene exon 4, 5, 6 circulation, and was found to be significantly decreased in plasma of MDD patients 152. Huang et al. found that circRNA stromal antigen 1 and 2 (STAG1) play a crucial role in attenuating depressive-like behaviors. circSTAG1 can bind ALKBH5 to inhibit its nuclear entry, thereby changing the m^6^A modification of total RNA and increasing the level of m^6^A modification of RNA, including fatty acid amide hydrolase (FAAH) mRNA 135. The expression of circSTAG1 in the hippocampus of CUS depressed mice was significantly reduced, and the m^6^A modification of FAAH mRNA was changed by ALKBH5, which ultimately led to the depression phenotype. 345-395aa of ALKBH5 may be the region that binds to circSTAG1. This study revealed that circRNA regulates the process of m^6^A modification by combining to m^6^A modification enzyme.

### m^6^A-modified circRNA in physical processes

#### Myogenesis

Increasing evidences have shown that circRNAs are abundant in skeletal muscles during the differentiation myoblasts, and the global expression level of circRNA changes dynamically 153. Additionally, several circRNAs (such as circ-ZNF609 and circular RNA supervillin have been proved played an important role in the differentiation and development of skeletal muscle 154. Here, newly study has found that 581 circRNAs are differentially expressed between skeletal muscle C2C12 myoblasts and myotubes. These myogenic-specific genes, 91 miRNA and the top 30 upregulated circRNAs forming a regulating network with 239 edges. Among the 581 circRNAs, 224 circRNAs have been identified as having coding potential. In addition, the number of m^6^A motifs in 224 cicrRNAs with encoding potential was also determined. Totally, 44 cicrRNAs had an m^6^A motif, 43 cicrRNAs had two m^6^A motifs, and 137 cicrRNAs had three or more m^6^A motifs. Gene Ontology and Kyoto Encyclopedia of Genes and Genomes analyses were performed that 75 cicrRNAs based on the linear counterparts were related with actin cytoskeleton and metabolic pathways 25. These annotations may offer novel insights in the development and differentiation of skeletal muscle and provide new therapeutic strategies foe muscular diseases.

#### Spermatogenesis

Spermatogenesis is a highly complex and specialized cell differential process during which diploid spermatogonial stem cells produce spermatozoa 155. Studies have shown that depletion of m^6^A writers (*Mettl3* and *Mettl14* or their homologs) can impair gametogenesis in multiple organisms 156. Recent research demonstrated that m^6^A is dynamically regulated and plays an important role in shaping gene expression during spermatogenesis 157. Moreover, a large number of circRNA will be generated from the thick line stage after meiosis to the round cell stage and the corresponding linear mRNA expression decreases during spermatogenesis 26. Tang et al. 26 studied the developmental process of male germ cells in mice that m^6^A modification can promote the formation of ORF-carrying circRNA. Sequencing analysis during spermatogenesis has found that a large number of circRNAs were generated while the corresponding linear mRNA was decreased. Some circRNAs often have high levels of m^6^A modification on both sides of the reverse junction point, and m^6^A is often enriched around the ORF start and stop codons of the mRNA. Interestingly, Tang et al. 26 found that nearly half of these circRNAs carry longer ORFs whose start codon is modified by m^6^A to bind ribosomes. Hundreds of peptides encoded by these circRNAs were detected by liquid chromatography and mass spectrometry. This research not only proved that m^6^A can mediate the generation of circRNA, but also discovered a novel mechanism for stable expression of circRNA-dependent protein product after linear RNA deletion.

## Conclusions and future directions

Recent researches on circRNA with m^6^A modification uncovered that epigenetic modification can also affect the circRNAs involved cellular process. CircRNA interacts with m^6^A-related proteins can regulate the progression and development of cancer. There are very few studies about circRNAs with m^6^A modification, so more understanding about the regulation and functions of this modification in circRNAs is still needed. Furthermore, m^6^A is most common in linear RNA and circRNA is considered to be the shaper of the RNA world 158. These findings indicate that the m^6^A modification may be a means for circRNA to achieve this reshape function. Therefore, m^6^A modifications in circRNA can uncover more information about the function of epigenetic modification in RNA world. However, limitations still exist in current researches. Further exploration can be conducted from the following aspects: 1) Determining the molecular mechanism of circRNA m^6^A modification (Are the 'writers', 'erasers' and 'readers' completely consistent with linear RNA?); 2) Elucidating the formation of cell types or tissues specific circRNAs (Why do circRNAs have specific modifications? Do they have a special function? Are there circRNAs that are not modified by m^6^A?); 3) Differentiating between specific exons and classical exons with m^6^A modification. Further studies are needed to unravel those mysteries.

Furthermore, this review describes the role, mechanism and application of m^6^A-modified circRNA in the pathophysiological processes. The most important role of circRNAs is in the development of various diseases and life processes. Nevertheless, the exact mechanism for m^6^A-modified circRNA in life progression is still unclear, because m^6^A modification can act as a double-edged sword. In spite of epigenetically modification play a significant regulatory role in the biological function of cell, while the terminal aim of medical researches is to be applied in the clinic. Designing targeted therapeutic drugs based on these newly elucidated molecular mechanisms the subject.

## Figures and Tables

**Table 1:** The mechanism of m^6^A-modified circRNAs in pathological processes

Type of disease	circRNA	Mechanisms of action	Putative biological function	Refs
Gastric cancer (GC)	circPVRL3	miRNA sponge or template for translation	Proliferation and migration of GC cells	129
Poorly differentiated gastric adenocarcinoma (PDGA)	A set of circRNA (has-circRNA-0077837)	-	Progression of PDGA	130
Colorectal cancer (CC)	circNSUN2	Protein scaffold (forms a circNUSN2/ IGF2BP2/ HMGA2 RNA-protein ternary complex)	Liver metastasis of CC	128
Hepatocellular carcinoma (HCC)	circRNA-SORE	miRNA sponge (miR-103a-2-5p and miR-660-3p)	Sorafenib resistance of HCC	132
Esophageal squamous cell carcinoma	circSLC7A5	Template for translation	Biomarker for cancer diagnosis and prognosis	140
Cervical carcinoma	circE7	Template for translation (E7 oncoprotein)	Link to the transforming properties of some human papillomaviruses	143
Acute coronary syndrome	Has_circ_029589	-	Induce macrophage pyrptosis	146
Hypoxia mediated pulmonary hypertension	A set of circRNAs (circ-Xpo6, circ-Tmtc3 )	Affect the circRNA-miRNA-mRNA network	Potential therapy targets	147
Major sepressive disorder	circSTAG1	Protein partner (Binding with ALKBH5)	Depressive	135

## Abbreviations

CircRNAs: Circular RNAs
ncRNA: Noncoding RNA
m6A: N6-methyladenosine
miRNA: MicroRNA
EcircRNAs: Exonic circRNAs
CiRNAs: Intronic circRNAs
EIciRNA: Exon-intron circRNAs
Pol II: RNA polymerase II
RBPs: RNA-binding proteins
QKI: Quaking
F-circRNA: CircRNAs derived from fusion-gene
ROS: Reactive oxygen species
SLC34A2-ROS1: Solute carrier family 34 member 2 and ROS proto-oncogene 1
UTR: Untranslated region
rt-circRNA: Read-through circRNA
HCV: Hepatitis C virus
NMVCs: Neonatal mouse ventricular cardiomyocytes
CRC: Colorectal cancer
ANRIL: The antisense ncRNA in cyclin-dependent kinase 4 inhibitor locus
INK4: Cyclin-dependent kinase 4 inhibitor
ARF: Alternative reading frame
SnRNP: Small nuclear ribonucleoproteins
MBL: Muscleblind
circMbl: Muscleblind circRNA
PABPN1: Poly(A) binding protein 1
HuR: Hu-antigen R
MDM2: Mouse double-minute 2
AML: Acute myeloid leukemia
FLT3: FMS-like tyrosine kinase-3
HDV: Hepatitis delta (D) virus
IRESs: Internal ribosome entry sites
ORFs: Open reading frames
SHPRH: Sucrose non fermenting 2 (SNF2) histone linker PHD RING helicase
LINC-PINT: Long intergenic non-protein-coding RNA p53-induced transcript
RFWD2: Ring finger and WD repeat domain 2
CTCF: CCCTC-binding factor
HHR: Hammerhead ribozyme
MTC: Methyltransferase complex
METTL: Methyltransferase-like
WTAP: Wilms Tumor 1 associated protein
ZC3H13: Zinc finger CCCH domain-containing protein 13
SnRNA: Small nuclear RNA
FTO: Obesity-associated protein
ALKBH5: α-ketoglutarate-dependent dioxygenase alkB homolog 5
YTH: YT521-B homology
IGF2BP: IGF2 mRNA binding proteins
mRNP: Messenger ribonucleoprotein
MRP: Mitochondrial RNA-processing
NSUN2: NOP2/Sun RNA methyltransferase family member 2
IGF2BP2: Insulin-like growth factor 2 mRNA-binding protein 2
HMGA2: High mobility group AT-hook 2
GC: Gastric cancer
PDGA: Poorly differentiated gastric adenocarcinoma
CC: Colorectal cancer
HCC: Hepatocellular carcinoma
circPVRL3: circRNA poliovirus receptor-related 3
ESCC: esophageal squamous cell carcinoma
hESCs: Human embryonic stem cells
RIG-I: Retinoic acid-inducible gene-I
PKR: Protein kinase
SLE: Systematic lupus erythematosus
PBMC: Peripheral blood mononuclear cells
HPV: Human papillomavirus
AS: Atherosclerosis
HPH: Hypoxia mediated pulmonary hypertension
ARC: Age-related cataract
ARCC: Cortical of ARC
LECs: Lens epithelium cells
MDD: Major depressive disorder
STAG: Stromal antigen 1 and 2
FAAH: fatty acid amide hydrolase
RNase: Ribonuclease
LM: Liver metastasis
RNPs: Ribonucleoprotein particles
NSCLC: Non-small cell lung cancer
